# Two Cases of Petechiæ; One Apparently Idiopathic, the Other Occurring in Typhous Fever

**Published:** 1819-09

**Authors:** Joseph Rogerson

**Affiliations:** Member of the Royal College of Surgeons, London.


FOR THE LONDON MEDICAL AND PHYSICAL JOURNAL.

### *Two Cases of Petechiæ; one apparently Idiopathic, the other occurring in Typhous Fever.* By JOSEPH ROGERSON, Member of the Royal College of Surgeons, London.

CASE I.—Mary Hopwood, ætat. 9 years, of a delicate constitution, spare habit, fair complexion, large blue eyes with dilated pupil, for the last two years has scarcely ever been well: the least exposure to cold, or irregularity in diet, brought on slight pulmonary affections. During the last few days she has frequently complained of languidness, with sickness at stomach; but the complaints were so slight as to be considered undeserving of notice: indeed, they were rather looked upon as favourable omens; for an habitual cough, which had troubled her from infancy, entirely ceased on the supervention of these symptoms. She rose this morning (Feb. 17) apparently in as good health as on the preceding day. In dressing, a few spots were observed upon her body, which, to use the expression of her friends, so nearly resembled flea-bites, that no further notice was at that time taken of them. In the course of the day these maculæ became more numerous, the breast and neck being most affected. When going to bed she was seized with a fit of vomiting, and threw up a considerable quantity of chocolate-coloured fluid, mixed with some clotted and some fluid blood. This recurred several times during the night; but the matter ejected had not the same colour, assuming a more florid red, not unlike arterial blood. In the morning the petechiæ were scattered over various parts of the body; and in the forenoon a further eruption appeared, accompanied with hæmorrhage from the nose, to such an extent as to induce fainting. During the last few hours, she altered so rapidly, and the symptoms increased with so much violence, that her friends became alarmed, and desired I would see her. This was early in the afternoon; but was not able to visit her before evening, when I found her recovering from a violent attack of vomiting, and blood was then slowly distilling from each nostril.

On examining her body, I found the petechiæ most numerous on the head, abdomen, and neck; the latter part they completely encircled. On the face they formed clusters of different sizes; on the hairy scalp there were a few, and these were larger than on any other part. On the thighs and arms they were also rather numerous; but, below the knees and elbows, very few. On the posterior parts of the body scarcely any were discernible, except a few across the loins, and three or four on the nates. They were of various magnitude, the largest not equal to that of a sixpence. Their colour was a light red, not at all resembling the dark purplish brown we sometimes meet with in the last stage of typhus. On passing the hand over their surface, some were prominent, others not in the least elevated.

She complains of severe pain across the forehead, general languor, oppression at stomach, with inclination to be sick; thirst not very considerable; tongue of a light flesh-colour; appetite not much impaired; skin hot and dry; bowels constipated; pulse upwards of 100 in the minute, full and intermitting. She was in a small close room; out of which I directed her to be removed into one where a constant ventilation could be kept up, by a current of air constantly entering from the window and door; to abstain from all solid food, and to drink, as her common beverage, barley-water acidulated with lemon-juice; and gave her ij grains of hyd. submuriat. with iij grains of pulv. antimon. to be taken every two hours; also a saline draught, containing eight drops of tinct. digitalis.

The following morning (February 18th) I found her much worse. She had passed a very restless night; the vomiting blood has been frequent; breathing is short and hurried; thirst immoderate; pulse scarcely perceptible; skin hot and dry, and conveys a peculiar stinging sensation to the touch; pupil insensible to the stimulus of light, neither contracting nor dilating on the approach of a candle; starts in her sleep, frequently tossing about her arms, and, when raised by an apparently unnatural effort, they are suffered to fall by their own gravity in any position; at the same time talking incoherently; and then sinks for a time into a kind of stupor, from which she is roused with difficulty; then answers questions put to her correctly, but in a rapid and petulant manner. Whenever she attempts to swallow, she complains of a sense of suffocation; in consequence of which only four doses of the medicine had been administered: the two latter were immediately rejected. The number of petechiæ are increased, especially on the back and arms. On inspecting the mouth, the tongue was covered with a dark-brown fur, its apex and sides were studded with petechiæ; also several large and distinct ones on the palatum molle, and as far down the throat as could be seen. Bowels had not been moved: I therefore requested an enema to be injected immediately, the powders to be given every hour, and the body frequently sponged with cold vinegar and water. The stomach being in so very irritable a state, I desisted from the use of foxglove, and desired nothing might be given which could be avoided. I left my patient, fully persuaded in my own mind she could not survive many hours; she being indeed then, to all appearances, *in articulo mortis*, and desired her friends would let me know immediately on this event taking place.

19*th*.—Contrary to my expectation, this morning I found the little patient much improved. The glyster had not been given, but the powders had operated copiously upon the stomach and bowels: an evacuation was procured by taking two doses of the medicine, and this action had been constantly kept up by their punctual administration; for the relief was so immediate, that even her attendants were convinced of their utility, and this induced them steadily to persist in the prescribed means. At first, the stools were in appearance not unlike the matter first ejected by vomiting, and very offensive: their colour gradually became redder, and the fætor has entirely subsided; still however they are tinged with blood. Oppression at stomach much relieved; vomiting ceased. Bleeding from the nose continues to recur, but at much longer intervals; breathing free; pulse 90, firm and regular. A little moisture has begun to appear upon the skin within the last hour, but the peculiar stinging sensation still continues. Tongue much the same as yesterday; thirst unabated; petechiæ unaltered, except upon the breast some appear shrivelled. She is quite cheerful, and expresses herself free from pain, but excessively weak. The cold ablution is so grateful to her she asks for its continuance; I therefore directed it to be used *ad libitum*, and that she should take the medicine only every four hours.

*20h.*—Has slept several hours very comfortably, and is in every respect much better. Thirst abated; pulse 90; head-ach removed; no return of sickness; bowels purged, stools slightly tinged with blood. Appearances were so favourable, that the cold washing was restricted to three or four times in the day, and to take a powder only every two or three hours. She expressed a wish for animal food, and was indulged with a small quantity without any unpleasant effect. In consequence of her living at a considerable distance in the country, I did not consider it necessary to see her any more: her friends therefore promised to bring me daily intelligence of the state of her health.

*24th.*—Up to this time my notes contain no other information, than she continues to improve. On this day they report her comparatively well; appetite good; no thirst; petechiæ almost disappeared, a few only remaining on the thighs and abdomen; sleeps well; no sickness; the saliva is slightly tinged with blood, owing in all probability to their still existing some maculæ within the mouth. Cold bathing has been discontinued for two days; but she persists in taking one of the calomel powders three times in the day, by which means several evacuations are daily procured; and, so great is the relief they afford, she invariably expresses herself "made better," and begs for a fresh supply. No other medicine was given, and the girl is now perfectly well.

In denominating this a case of petechiæ, I am aware that objections may be urged against it, from the opinion that petechiæ can only result from a general deranged action of the vascular system; and that the previous symptoms had not evinced such a state of the sensorium, as sufficiently accounted for that derangement. Though the previous indisposition was too trivial to engage the attention of her friends, I cannot positively assert it did not amount to febrile excitement; but I am not disposed to consider the action sufficiently violent, or long enough continued, to induce that debilitated state of constitution which must ensue, before the eruption, from that cause, could take place. On the 17th, febrile symptoms increased with the fresh appearance of petechiæ; and, in proportion as these maculæ became more numerous on the two succeeding days, constitutional derangement was aggravated. Hence I am induced to believe, that, in this instance, the febrile symptoms were rather an effect than a cause of the petechial eruption. This is an opinion I am not anxious to contend for on account of its novelty, but because the inferences deducible from this view of the subject will lead us to correct treatment, and enable us to arrest the progress of a complaint which heretofore has most generally terminated fatally; for, whether the action be spontaneous and induce the disease, or the disease primarily exists and excites the febrile affection, the proper line of conduct is evidently to diminish vascular action; but, according to the relative proportion in which action and debility are combined, may we be more or less energetic in the measures employed for this purpose.

Whilst pursuing my professional studies, I had an opportunity of seeing several cases of petechiæ *sine febre,* where the usual remedies were employed. Some of them terminated fatally; and in those which recovered, under a liberal use of the mineral acids, the pulse was invariably reduced below the healthy standard, both in frequency and strength. This made me determine to practise more decidedly, should another case come under my care. Conscious that, in the present instance, prompt and decisive measures were necessary, I adopted the plan recited; but omitted the foxglove on the 18th, owing to the irritable state of stomach, which I supposed it contributed to keep up. I was strongly tempted to employ the lancet; but, a slave to preconceived opinions, and apprehensive of the fatal consequences which I feared would follow its use, I had not resolution enough to call in its aid. From the favourable termination of this case under the means instituted, we are warranted in concluding they were beneficial; and I am disposed to think, had more active doses of purgative medicine been given, so as to produce an early effect on the bowels, it would have prevented the state in which my patient was the following day.

It is highly probable, more immediate relief would have been obtained by blood-letting; but this is a practice, the efficacy of which remains to be fully ascertained, and can only be done by repeated trials; for since, in the absence of facts, abstract reasoning is at least questionable, we must wait until public experience either sanctions or condemns a remedy, which, if used with discrimination, promises fair to alleviate the sufferings of mankind.

The fatality most commonly attending these cases, induced me to communicate one, where the advantages of the practice detailed was obvious, without waiting perhaps a number of years to accumulate facts enough to decide on the propriety of such practice in general. If practitioners who have had opportunities of treating this complaint, would impart the result of their experience, the question would soon be set at rest.

Case II.—Thomas Waring, ætat. 15, living in a crowded part of the town inhabited principally by poor half-starved families, and from whence fever had not been absent for several months, had been ill twenty-one days before I saw him; which I was requested to do, in consequence of a copious eruption of petechiæ over the whole body. They were first perceived yesterday, upon the breast and face; to-day, they have made their appearance on other parts; and now (April 6th) no part is exempt from them. They are of a dark-purplish colour, and various sizes; none of them, however, larger than a pea.

He is quite insensible, occasionally muttering something incoherent; tongue coated with a dark-brown fur; the teeth and their interstices are likewise covered with the same matter; breath offensive; thirst excessive; bowels costive; pulse, I know not how to describe better than by saying, it conveyed to the finger a sensation similar to a continued stream of water passing through an elastic tube. He frequently applies his hands to his head, as if conscious of pain. Has voided bloody urine several times since the attack. During his illness he has taken an occasional purgative only.

I took twelve ounces of blood from his arm, when he fainted. On his recovery the pulsation of the artery could be distinctly felt beneath the finger; but it beat with so much rapidity and irregularity, it could not be counted. A powder, containing vj grains of jalap and iij of calomel, was ordered to be taken every hour; and the body directed to be frequently sponged with cold vinegar and water.

*7th.*—Powders have operated freely, producing many stools of a dark colour, and intolerably offensive. Pulse 120, feeble, but quite distinct; perfectly sensible, and complains of oppression at his chest, and acute wandering pains in his head; thirst very considerable; tongue same as yesterday; no evident alteration in the eruption; skin not so hot and dry. He expresses himself much refreshed by the cold-bathing, which, with the medicine, was ordered to be continued as before.

*8th.*—Has had some comfortable sleep in the night, and perspired rather profusely. Thirst much abated; the tongue begins to assume a yellowish appearance; pulse 120; bowels continue open; stools of a light-red colour, and much less offensive; petechiæ not so numerous on the thighs and abdomen. This morning, feeling an inclination to eat, his mother indulged him with a small piece of toast, and a basin of tea: this produced great uneasiness at stomach, which was only relieved by vomiting. I prohibited his taking any more solid food. Continue medicines and the cold ablution.

*9th.*—Continues better. Pulse 100; tongue almost free from fur; bowels purged, and the dejections neither unnatural in colour nor smell. He complains of the medicine griping; to prevent which, I combined about 1-6th of a grain of opium with each powder, and desired him to take one every three hours. Thirst almost gone; petechiæ begin rapidly to decrease in number, especially upon the upper and lower extremities.

*10th.*—Considerably better; has slept the whole of last night. The medicine procured four evacuations from the bowels; griping ceased; pulse below 100; no thirst; skin cool; has eaten some bread and milk, without producing any uneasiness. A few petechiæ remain on the breast, back, and abdomen; all the others have entirely disappeared. The cold affusion was only used twice yesterday.

*11th*, *12th*, *13th*, and *14th.*—Continues to improve; petechiæ entirely disappeared. The medicine was persisted in until today, the quantity having been gradually diminished. Appetite is now very good, and the only symptom to contend against is debility. The cold washing has been discontinued for two days. He now takes freely of animal food, and is allowed a pint of porter daily.

From this time his convalescence was rapid; in a few days he was able to take exercise out of doors, and is now perfectly well.

The exceeding prevalence of the late epidemic fever has fully proved the utility of blood-letting, and other depletory measures, in its early stage; whilst it has afforded innumerable facts corroborative of Dr. Clutterbuck's theory, and established the propriety of the treatment instituted by him, and other eminent medical characters. It has also tended in no small degree to dispel the mists of prejudice which have so long enveloped the minds of medical men, and thus prevented its being brought into general use. Its advocates have only now to fear it will be brought into disrepute, by incautious and ill-timed application,

Although ts efficacy is admitted by most in the beginning of fever, there are few who would subscribe to its employment in the latter stage; nor should I wish to be understood as advocating the use of the lancet in every case; for I am well aware, that a rash and indiscriminate perseverance, would be a more dangerous practice than refraining from it altogether.

In cases, however, where we have a train of symptoms to contend against, arising from excessive circulation in some important organ, which, if not speedily removed, would soon terminate fatally, and where few would hesitate to pronounce purgatives were the only medicines from which we could expect success, because their efficacy consists in the depletory effects they produce, removing irritation, and equalizing the distribution of blood; is it not better immediately to effect our purpose by the abstraction of blood, rather than wait the more tardy operation of medicine, which could achieve only the same end, and cause a delay by which we might lose our patient?

In this case, I should consider petechial effusion (if I may so term it) as a consequence of increased vascular action in a constitution already debilitated by disease; for, the greater the debility, the less increase of action will be necessary to produce this effect; and this exhausted state of constitution might be urged against adopting a measure which would tend still further to weaken our patient. To this I would reply, the loss of a small quantity of blood, sufficient to produce the desired effect, would not bring on prostration of strength adequate to what would ensue from a continuance of the disease.

Appearances in Waring's case were so unfavourable, I was apprehensive lest, in waiting the operation of purgatives, the complaint would increase, and my patient shortly be in a state over which human means could have no control: it is in similar instances, and as a remedy which ought never to be lost sight of, I would recommend blood-letting. The good effects of this practice were immediately evident, and certainly such as to warrant a repetition. In the more advanced stages of fever, when the lancet was not admissible, I have seen much benefit derived from topical bleeding with leeches.

It is not the least remarkable of the phenomena of fever, to what a considerable extent active depletory measures may be carried, even in poor half-famished families, (among whom the complaint has principally prevailed in this town,) and yet produce so little subsequent debility. If the patient has applied early in the disease, its progress has generally been arrested by brisk purging with calomel and jalap, or a solution of neutral salts in an infusion of senna, and ipecacuanha wine, the action of the bowels being kept up without intermission for three or four days; at the end of which time all complaint has ceased, and the person has generally been able to return to his usual occupation, expressing himself stronger than before his illness. If the complaint has existed for some time before application is made for relief, the period of convalescence has commonly been long and protracted.

*Wigan; July 9th,* 1819.

